# METASEED: a novel approach to full-length 16S rRNA gene reconstruction from short read data

**DOI:** 10.1186/s12859-024-05837-z

**Published:** 2024-07-12

**Authors:** Melcy Philip, Knut Rudi, Ida Ormaasen, Inga Leena Angell, Ragnhild Pettersen, Nigel B. Keeley, Lars-Gustav Snipen

**Affiliations:** 1 Faculty of Chemistry, Biotechnology and Food Science, Norwegian University of Life Sciences, Ås, Norway; 2 Akvaplan-Niva, Tromsø, Norway; 3 Institute of Marine Research, Tromsø, Norway

**Keywords:** 16S rRNA, Metagenomics, Seafloor sediment, Taxonomic profile

## Abstract

**Background:**

With the emergence of Oxford Nanopore technology, now the on-site sequencing of 16S rRNA from environments is available. Due to the error level and structure, the analysis of such data demands some database of reference sequences. However, many taxa from complex and diverse environments, have poor representation in publicly available databases. In this paper, we propose the METASEED pipeline for the reconstruction of full-length 16S sequences from such environments, in order to improve the reference for the subsequent use of on-site sequencing.

**Results:**

We show that combining high-precision short-read sequencing of both 16S and full metagenome from the same samples allow us to reconstruct high-quality 16S sequences from the more abundant taxa. A significant novelty is the carefully designed collection of metagenome reads that matches the 16S amplicons, based on a combination of uniqueness and abundance. Compared to alternative approaches this produces superior results.

**Conclusion:**

Our pipeline will facilitate numerous studies associated with various unknown microorganisms, thus allowing the comprehension of the diverse environments. The pipeline is a potential tool in generating a full length 16S rRNA gene database for any environment.

**Supplementary Information:**

The online version contains supplementary material available at 10.1186/s12859-024-05837-z.

## Background

Over the past few decades, full-length 16S ribosomal RNA (rRNA) gene sequences have become the core of microbial taxonomy [1, 2]. Moreover, with Oxford Nanopore Technology, the sequencing of full-length 16S amplicons from environmental samples is feasible, and because of the portability of this technology, on-site monitoring of the environment is possible [3]. Due to the error-prone nature of such data, full-length 16S reference sequences are needed for data analysis [4, 5]. However, obtaining high-quality full-length sequences from microorganisms that cannot be cultured is a major challenge, as most such data are still obtained by short-read amplicon sequencing. Although, in theory, shotgun metagenome data should enable the reconstruction of full-length 16S rRNA gene sequences [6] the Metagenome Assembled Genomes (MAGs) often lack, or have poor quality, 16S rRNA genes [7].

Despite advancements in long-read technologies [8], the primary challenge in analyzing environmental DNA still lies in issues such as sample purity and fragmentation, which render long-read metagenome sequencing difficult or impossible [9]. The error-prone nature of long reads and the difficulty in scaling data analysis tools to handle the data produced by long read technologies also remain challenges [10]. Hence, developing strategies for building high-quality full length 16S rRNA gene sequences from short read DNA sequencing data, originating from environmental samples would be beneficial.

There have been some efforts to develop tools for reconstructing the 16S rRNA gene from metagenome data, such as Emirge (Expectation–Maximization Iterative Reconstruction of Genes from the Environment) [11] and MATAM (Mapping-Assisted Targeted-Assembly for and Metagenomics) [12]. They use a mapping-based approach to identify, align, and assemble metagenome reads, using a collection of already known full-length 16S sequences as a reference. MATAM focuses on generating profiles based on the existing full length 16S rRNA gene sequence databases and has been successful in doing so. However, the opportunity to comprehend and create novel long 16S rRNA genes is still lacking.

**Fig. 1:**
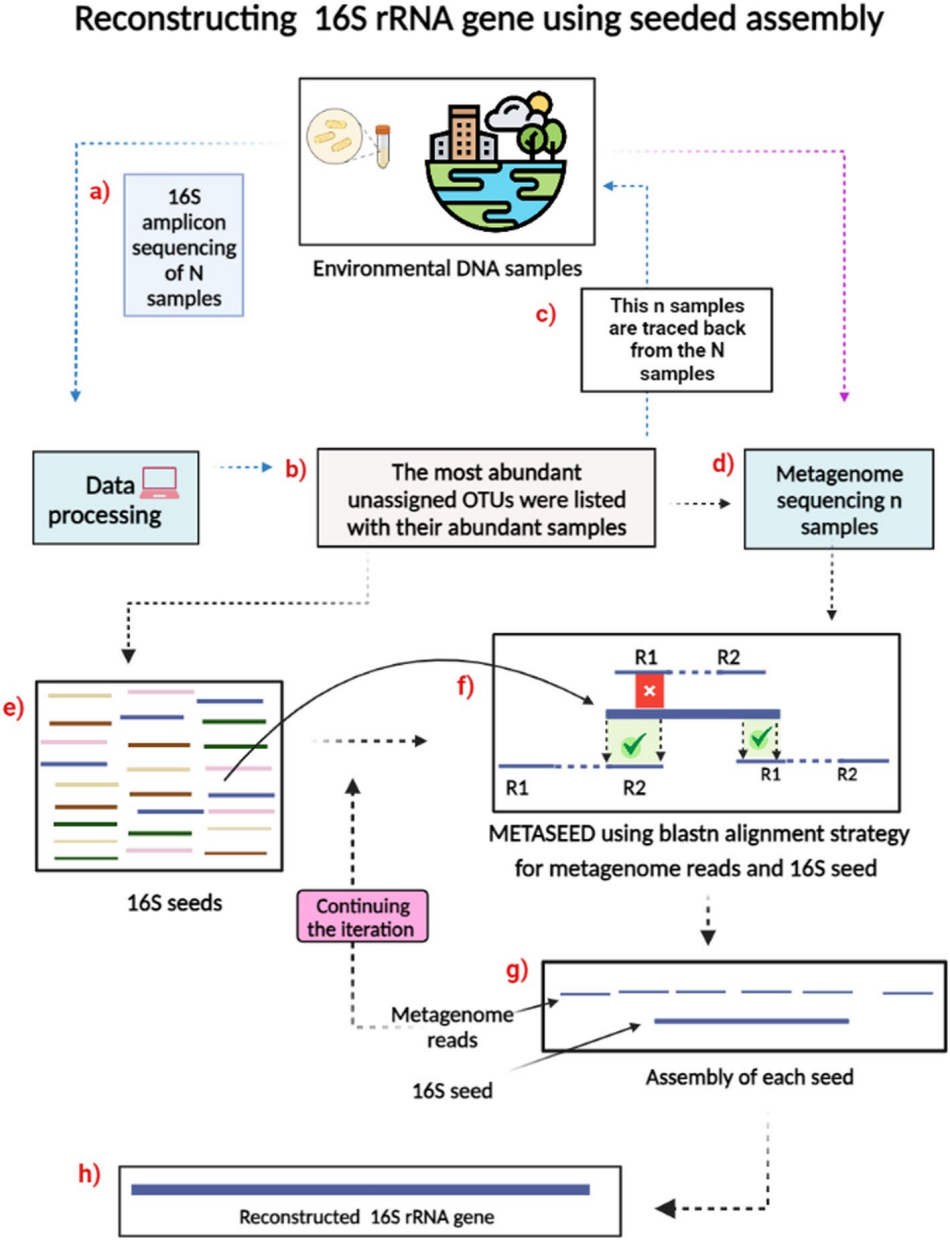
The illustration depicts the process of METASEED, which utilizes both amplicon and shotgun data from the same sample set. Box **a** shows how we started with N samples of 16S amplicon sequencing data. In box **b**, the most abundant OTUs with no database match are listed along with the samples where they are most abundant. In box **c**, **d**, these n samples are then subjected to metagenome shotgun sequencing. The 16S seeds were selected from the n samples in box (**e**). In box (**f**), the seeds are matched to the metagenome reads under specific conditions and reads that matched either the entire seed or the end of the seed were selected for further analysis (green tick), while the partially matched reads were discarded (red tick). Box **g** shows the assembly of each seed using the SPAdes assembly tool to produce near full-length 16S rRNA genes box (**h**). Created with BioRender.com

In this paper, our aim is to develop strategies to reconstruct full length 16S rRNA sequences when there is a lack of a reference sequence in public databases. Our strategy is based on the use of short-read amplicon 16S rRNA gene and full metagenome sequencing data from the same samples. This method is designed to broaden the repertoire of sequences in reference databases by reconstructing novel near full length sequences. This means that based on the 16S amplicons, we have very precise information about how a smaller region of the 16S genes in our samples look. Using these datasets, we propose an alternative method called METASEED as shown in Fig. 1, where these amplicon sequences are considered ‘seeds’, the matching metagenome reads are collected, and from these, we attempt to reconstruct (or grow) a larger part of the 16S gene for each seed separately. Seeded assembly may improve the accuracy and completeness of assembled contigs, particularly when working with environmental data that are difficult to assemble due to high diversity and fragmented DNA.

## Results

### Simulated data

A key to the success of our approach lies in the ability to collect the best possible set of metagenome reads for each of the seed sequences. On the one hand we need to collect enough read pairs to be able to assemble and extend the seed into a near full-length 16S, but on the other hand, collecting too many incorrect reads, i.e., reads that do not truly come from the 16S of the seed, will turn the assembled sequence into a mosaic of little value. To investigate the potential pitfalls here, we started with simulated data. This allows us to annotate every single read with the exact genome from which it originated, facilitating us in assessing how many correct and incorrect reads were collected for each seed, given necessary filtering options.

**Fig. 2:**
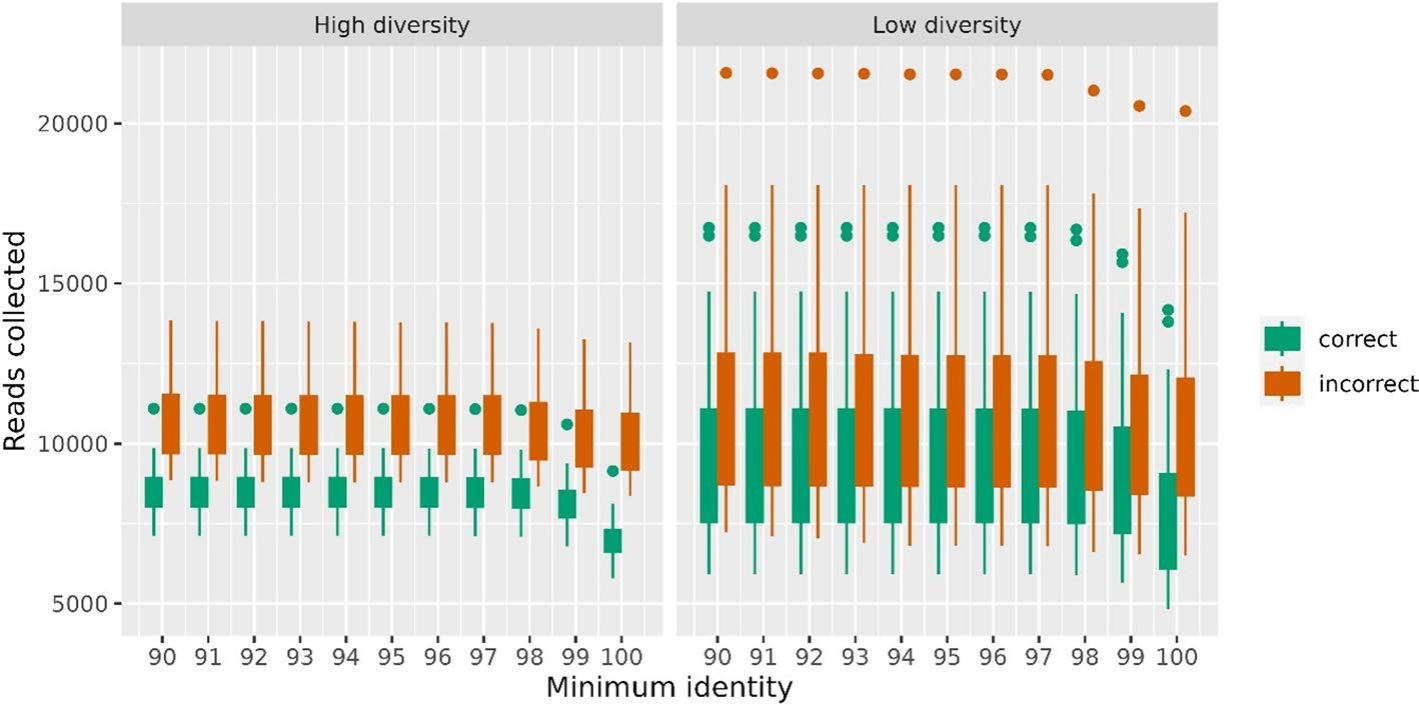
The effect of sequence identity on the collection of metagenome reads matching the seeds. Different minimum identity thresholds (percent) were tested (x-axis) and the numbers of correctly and incorrectly collected reads were counted for all seeds in all 40 simulated samples. All metagenome samples contained 10 million read pairs. The green and red boxplots at each minimum identity indicate the spread between the samples. The left panel shows 20 high-diversity samples, while the right panel shows for 20 low-diversity samples

Figure 2 depicts the variation in the number of reads collected correctly and incorrectly with respect to the minimum alignment identity. Note that since in the simulated data, all reads are tagged by which genome it originated, making it possible to see if a read is ‘correct’ or ‘incorrect’ with respect to the seed it matches. Apparently, more incorrect reads align with the seeds than with correct reads, and when the identity surpasses 98%, there is a decrease in the number of correct reads. Thus, a 98% identity threshold was used below unless otherwise stated. We also notice the number of reads that aligned to the seeds for low -diversity samples was greater than that for high-diversity samples.

**Fig. 3:**
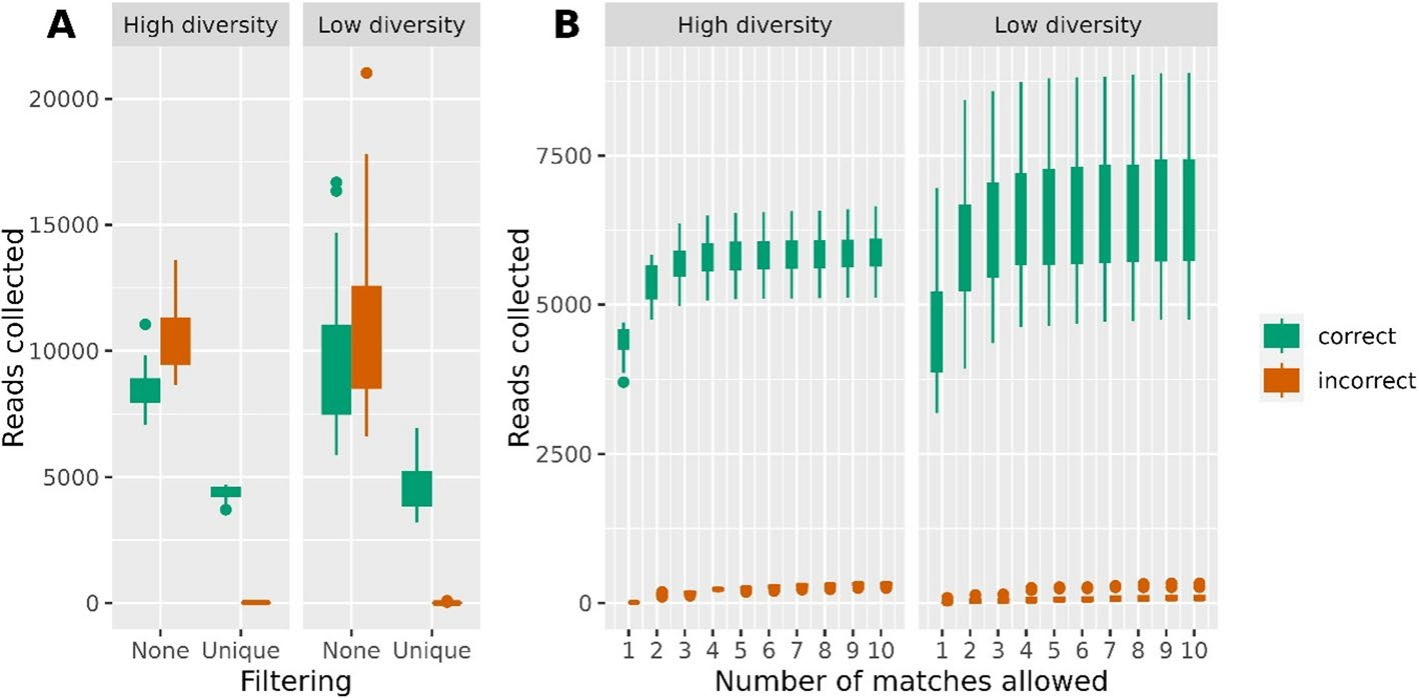
The number of correctly and incorrectly collected reads under various filtering regimes. In the left panel (**A**) we show the effect of collecting all reads (filtering None) versus only collecting reads who give a unique match against one single seed (filtering Unique). Again, we split the samples by diversity. In the right panel (**B**) we relax the uniqueness by allowing reads to match from 1 up to 10 different seeds, but then assign the read to the seed with the largest abundance, based on the amplicon data. The result for 1 match in the right panel is identical to the uniqueness criterion in the left panel

We found that many reads matched two or more seeds equally well, a potential explanation for the results shown in Fig. 2. We then tried to collect only reads who aligned uniquely to one seed only. In the left panel of Fig. 3, we show the effect of employing this uniqueness criterion. A comparison of the boxplots for no such filtering (None) to requiring uniqueness (Unique) revealed a dramatic decrease in the number of incorrectly collected reads (red boxplots). However, we also observed a substantial decrease in correctly collected reads (green boxplots), indicating that this criterion is perhaps a bit too strict. Then we explored the relaxation of the uniqueness criterion by using the seed abundance information from the 16S rRNA gene data. If a read matches two or more seeds equally well, we always assign the read to the seed with the highest abundance in the sample. The logic is simply that the read is more likely to come from a more abundant seed, given that it matches several equally well. In the right panel of Fig. 3, we highlight how the number of collected reads increases by allowing reads to match up to 2, 3, …,10 different seeds. Allowing up to three matches, increases the number of correctly collected reads without significantly increasing the number of incorrect ones.

**Fig. 4:**
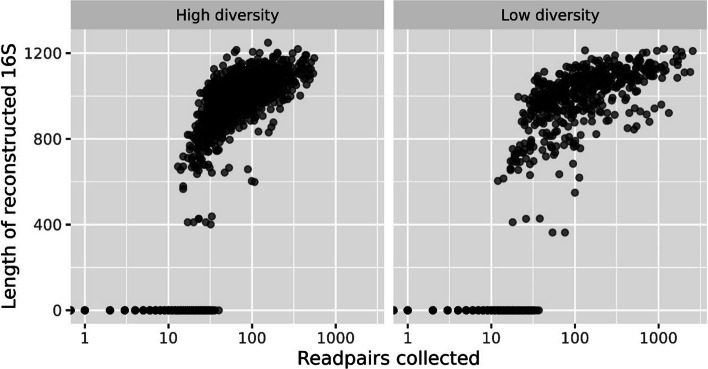
The number of read pairs required to assemble the 16S. Each dot is an assembled 16S contig from one of the samples of simulated data. Along the x-axis is the number of read pairs collected and the y-axis is the length of the resulting assembled contig. Contigs of length 0 indicate the assembly failed, and we observe this is the case when the number of read pairs decreases to less than approximately 40. Whenever a contig is assembled, its length tends to grow by the number of read pairs

After setting the criterion where we allow up to 3 matches, and assigning reads by seed-abundance, we collected the read pairs and assembled them for each seed and sample. By comparing the resulting reconstructed sequence to the 16S in the genomes from which we simulated, we found that by far most of the reconstructed gave an excellent match (> 99% identity, alignment covering > 99% of the sequence). In Fig. 4 we instead show how the number of collected read pairs affect the reconstruction of the 16S. It is obvious that we need more than 10 read pairs, and in most cases more then 40, to be able to reconstruct a 16S with some substantial length. As expected, the more (correct) reads we collect, the longer the reconstructed sequence.

### Metagenomic dataset of seafloor sediments

To demonstrate the 16S reconstruction on real data we started out with 16S amplicon sequencing (Illumina MiSeq) of 286 samples taken from seafloor sediments, an example of an environment with rather large diversity and with many ‘unknown’ 16S variants. Our 16S pipeline produced a total of 70,578 OTUs (99% identity clustering) from these data. A subset of 24 samples were then selected based on having amplicons not matching (> 99% identity) known 16S in the SILVA database [13]. These 24 samples cover a total of 9296 of the OTUs. The eDNA from these samples were then subject to full metagenome sequencing (Illumina Novaseq 6000). From this we selected the top 300 most abundant OTUs as our seed sequences, from which we want to reconstruct as much as possible of the full 16S. In real life, only the seed sequences not matching anything in the public databases would be of interest, but in this case, we included all the top 300 seeds regardless of this. This is because, it gives us preliminary indications about whether we reconstructed something comparable to what the full-length sequence in the public database suggests.

### Reconstructing 16S rRNA gene from amplicon and shotgun data using METASEED pipeline

**Fig. 5:**
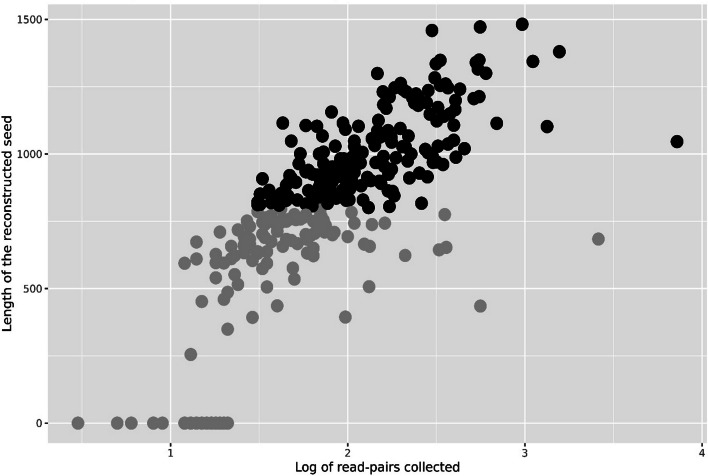
The figure shows the log number of metagenome read pairs (x-axis) collected for each seed (dots) along with length of the reconstructed 16S for each seed (y-axis). The black dots represent reconstructed 16S with a minimum length of 800 bp. Dots at zero length indicates seeds where no reconstruction was possible

We employed the strictness criteria learnt from the simulated data results above, i.e. we collect metagenome reads who align with at least 98% identity to some seed, and allow alignment against up to 3 different seeds, but then assign the read to the seed with largest abundance in the sample in question. In Fig. 5, we show the log of the number of read pairs collected, and the length of the reconstructed 16S for each seed. As we observed for the simulated data, the length of the reconstructed 16S sequence tended to increase as we collected more reads, with substantial variation. The METASEED resulted in 282 of the 300 seeds having a reconstructed sequence. Of these, 185 had a minimum length of 800 bp. We use this as a minimum length of interest, since this means it covers > 50% of the full length. Also, the seeds are themselves already 400–450 bases long. The chimera check showed that none of the reconstructed 16S were chimeric.

**Fig. 6:**
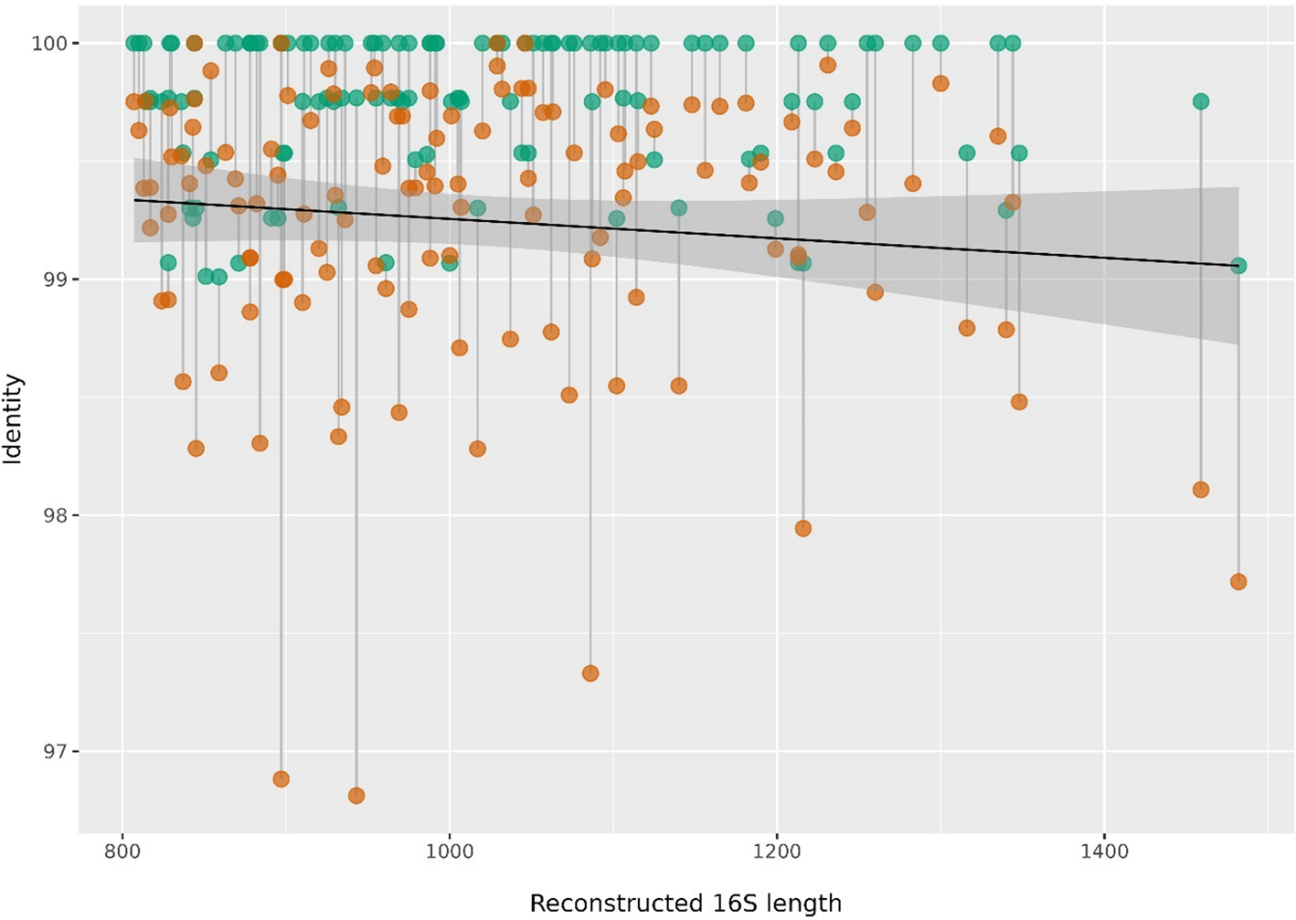
In total 125 seeds achieved a reconstructed sequence of at least 800 bases length and at the same time had a match (> 99%) against at least one full-length SILVA 16S sequence. The 125 green dots indicate the identity of this match (y-axis). The red dots indicate how well the corresponding reconstructed sequence matched the corresponding SILVA sequence. The location along the x-axis marks the length of the reconstructed sequence for each seed/reconstructed sequence pair. Additionally, the trend line indicates a very weak drop in identity for longer reconstructed sequences

Since we did not filter the seeds for already known 16S sequences, some of them matched full-length 16S sequences in the SILVA database. BLASTing the seeds behind the 185 reconstructed 16S against the SILVA database revealed a near-exact match (> 99% identity) for 125 of them. For a sanity check, these 125 seeds were also blasted against the corresponding reconstructed 16S sequences, to verify the reconstructed 16S sequences indeed had the best match to their corresponding original seeds. A second blast analysis using the corresponding reconstructed 16S sequences from these 125 seeds against the SILVA database, revealed that these also had the best match to the same sequences, or to a similar SILVA sequence of the same species, where the seeds matched. Figure 6 shows the BLAST identities for these 125 cases. The green dots indicate the seed, and the red dots represent the corresponding reconstructed 16S. The position along the x-axis for each green–red pair indicated the length of the reconstructed sequence. The figure demonstrates how well the seeds, and its reconstructed sequences matched the same SILVA sequence (colored dots) in terms of sequence identity. In addition, the trend line indicates there is little loss in identity for longer reconstructed sequences. We also tried to vary the uniqueness criterion when collecting reads, and the results of this are shown in supplementary Table S1.

### Alternative ways to obtain 16S rRNA gene from shotgun data

Once we have the metagenome data for the 24 samples, it is of course natural to assemble these and see if we find many 16S sequences in the resulting contigs. The assembly pipeline resulted in a large number of contigs for each sample, in total 26,133,575 contigs. We used the Barrnap software to search all contigs for 16S. This resulted in the detection of 195 bacterial sequences and 186 archaeal sequences. All these 381 sequences were non chimeric and above 800 bases in length. However, since we must expect several of these being 16S variants from the same species, the Barrnap sequences were clustered using VSEARCH with 99% identity. This resulted in 150 distinct 16S sequences. To see to what extent these are already known 16S sequences, we did a BLAST analysis of the Barrnap sequences against the SILVA database. From this we found that 128 matched something (> 99% identity). We then compared the Barrnap sequences to the OTUs from our amplicon data and found only 51 matches (> 99% identity). We then made a similar study based on our simulated data sets, since here we know exactly how the full-length 16S sequences look like. The simulated shotgun data were assembled in the same way as the real data, and Barrnap was used to collect 16S from the contigs. Of the 253 distinct 16S sequences found this way, only 1 gave a > 99% match to some of the actual 16S sequences, indicating very clearly that a short-read metagenome assembly of 16S easily becomes a mosaic of several sequences.

The *de-novo* assembly using the MATAM tool was performed on the 24-shotgun metagenome samples. In MATAM a contig of at least 500 bp is considered as a reconstructed 16S. The MATAM tool found 14 non-chimeric 16S sequences of this minimum length, the longest of which was 649 bases long.

### Comparing METASEED to existing tools

**Fig. 7:**
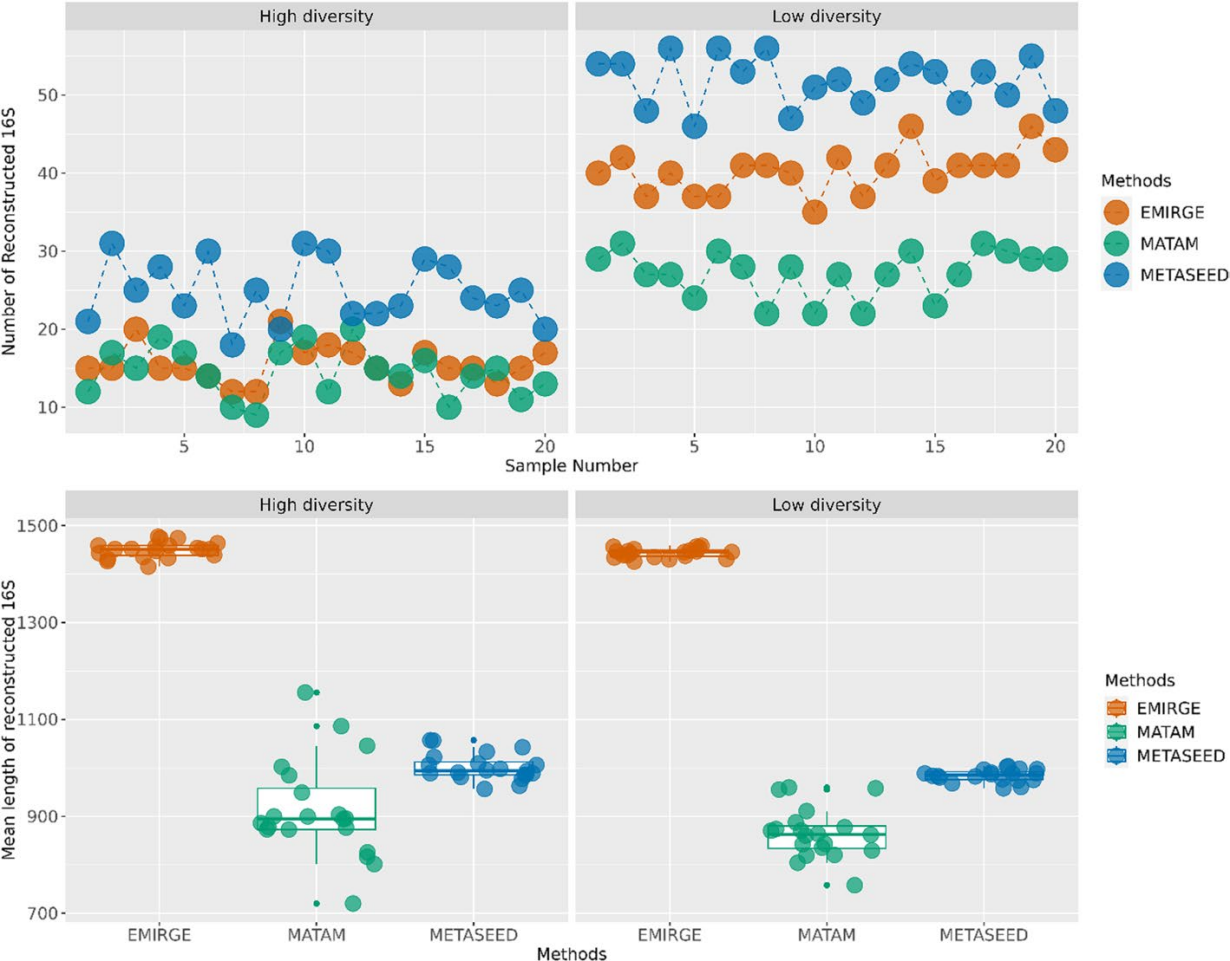
Displays the count and average length of reconstructed 16S sequences obtained from three methods, each with at least 99% identity to the actual 16S sequences across various samples. The upper panel illustrates sample numbers on the X-axis and the corresponding count of reconstructions on the Y-axis. The lower panel represents the methods utilized on the X-axis and the mean length of the reconstructed 16S sequences for each sample on the Y-axis. Different methods are denoted by color in both panels

We compared METASEED to EMIRGE and MATAM using the simulated data mentioned above. In total EMIRGE reconstructed 1589 sequences, MATAM 992 and METASEED 1631 from all samples. Comparing these to the actual 16S behind the data we required > 99% identity for count it as a successful reconstruction. This resulted in discarding 471 of the EMIRGE, 160 for MATAM and 97 from METASEED. In the upper panels of Fig. 7 we display the number of successfully re-constructed for the various methods in each sample. The lower panels show the length distribution of the successfully re-constructed sequences.

## Discussion

The reconstruction of the full-length 16S rRNA gene from short-read metagenome data is challenging, limiting the taxonomic exploration and analysis of environmental samples [14]. The fact that 16S is highly conserved across taxa imposes some limitations on the assembly of this gene from short reads [15]. Based on our simulation studies, as shown in Fig. 2, we observed that when aligning shotgun reads to the amplicons from the same samples, we collect a greater number of incorrect reads than correct reads, even with exact matching to the amplicons. This clearly demonstrates the problem faced here. In general, if we assemble all these collected reads (incorrect and correct), we are unlikely to reconstruct the original 16S. This is the reason for the poor or no reconstruction of 16S directly from metagenome reads [16]. Hence, we developed METASEED as an alternative where we use amplicon 16S rRNA data and shotgun sequencing data from the same samples, helping the pipeline to determine how the original 16S region would look.

After our multiple tuning trials, we found certain criteria that help in reducing the number of incorrect reads, and thus performing the assembly on the best possible data. As a first step, blastn parameters were investigated and optimized, as shown in Fig. 2. Often 16S analysis uses 97% identity as the threshold [17], but we found a 98% identity was optimal as the number of correct reads dropped beyond this scale. In general, we collect more reads from low diversity samples. This indicates that real environmental samples, often having a very high diversity, are the most challenging type of data to work with. Since many reads match many different seeds equally well, we regulated this by specifying the maximum number of seeds a read pair can match, before the read must be discarded. In Fig. 3, panel A, we illustrated the effect of uniqueness (number of matches = 1), where the number of incorrect reads then drops to nothing, but also causes a decrease in the number of correct reads. This essentially means we only collect reads matching the unique pieces of the 16S, which we suggest too strict for having data enough to reconstruct the full length 16S. To avoid this decline, the uniqueness criteria were then relaxed as seen in panel B of Fig. 3. Again we made use of the amplicon data, by allowing a read pair to match up to 3 distinct seeds, but then assigning it to the seed with the largest abundance in the sample. From the simulated data this seems to recover many correct reads, without appreciably increasing the number of incorrect reads. As we anticipated, Fig. 4 shows that we must collect a minimum number of correct read pairs to reconstruct high quality long 16S.

Next, we tested this approach on real data from seafloor sediments. From 24 samples where we have both amplicon and shotgun data, we selected the 300 most abundant OTUs as seeds, and used the METASEED to reconstruct as much as possible of the 16S surrounding these amplicons. From the 300 seeds we got reconstructions for 282, of which 185 were at least 800 bases long, as seen in Fig. 5. In the real data, we have no knowledge of the actual 16S in the samples. However, 125 of the 185 reconstructed sequences had a match to the SILVA database, which is at least some indication of correctness, since this sequence has been observed before. The trend line in Fig. 6 depicts the overall trend in the variation of identity for the seeds and their corresponding reconstructed 16S sequences to the SILVA sequences, indicating the quality of reconstructed 16S. Apparently, there are very few reconstructed 16S that has an identity drop below 96%. We also performed METASEED analyses using uniqueness criteria 2 and 1 as shown in Fig. 3, panel B. The idea being that a stricter read collection may be beneficial for real compared to simulated data. Seemingly, there was no improvement in the number and quality of the reconstructed 16S as displayed in supplementary table S1.

Tools such as EMIRGE or MATAM also try to re-construct the 16S from WGS reads, but instead of using amplicon data from the same samples, they rely on already known full-length 16S sequences. Although this setup differs from our setting, we did a comparison of these methods on the simulated data, wherein all genomes and 16S sequences included are from already known taxa. Still, our results in Fig. 7 indicate that METASEED is consistently capable of reconstructing more of the 16S in the shotgun data, even if the EMIRGE typically produces longer sequences. However, EMIRGE produced approximately thirty percent unsuccessful reconst ructions, i.e. a 16S sequence less than 99% identical to the actual true sequence, whereas METASEED only produced six percent such ‘false positives’. As expected, all methods perform better on low-diversity data, since this gives more coverage to the dominating taxa. Even if these results indicate the usefulness of METASED, we must stress that its real benefit lies in the potential to re-construct novel 16S where no reference is available, as required by the other tools.

We also used the software MATAM on the real data, but this resulted in very few 16S being reconstructed. EMIRGE was not tried on these data since it would take a very long time to run on these very large data sets. When compared to these tools, METASEED is also more efficient in terms of running time and computational resources.

Apparently, the most obvious approach to finding 16S is to simply assemble shotgun reads in the conventional way, and then use a tool like Barrnap to look for 16S in the resulting contigs. We also did this for the 24 sediment samples and found 150 distinct 16S sequences of length 800 or more. This is almost as good as METASEED, with 185 sequences. However, of these only 51 gave a good match to some of the amplicon OTUs for the samples, indicating two thirds of these are either mosaic sequences, or from taxa not amplified by our primers. When we used the same procedure on the simulated data only 1 out of 253 sequences collected this way matched one of the actual 16S sequences that we know are in the samples. We believe this indicates the 16S collected directly from metagenome assemblies are mostly mosaics that look like a ‘general’ 16S sequence but probably contains pieces from many different taxa. These are also difficult to reveal by conventional chimera checking, since the latter is designed to find mixtures due to PCR amplification, where the first and the last part of the sequence comes from distinctly different taxa. Here we most likely face mosaics of several taxa, with pieces intermixed along the sequence.

The METASEED sequences are based on an amplicon that was amplified and sequenced in high abundance in the same samples. This implies we start with a rock-solid fragment of the 16S for one taxon. The challenge then resides in collecting the appropriate shotgun reads associated with this seed sequence, hence the requirement for the fine tuning we present on this. Conventional metagenome assembly of environmental samples is typically performed sample by sample, rather than as a co-assembly, simply because the samples are often too distinct. However, with METASEED we collect 16S-matching reads to each seed across all samples before we start the assembly, which is then no longer a metagenome assembly but done for each seed separately. As a result, we get a significantly greater coverage of the 16S for each seed compared to if we limited ourselves to the (few) reads in each sample. To the best of our knowledge, this work builds on prior research in related domains while making a distinct contribution to the subject.

## Conclusions

In this study we have shown why reconstructing the 16S gene from short-read metagenome data is extremely challenging, even close to impossible, without some form of reference to start out with. In our proposed METASEED approach, we use amplicon data from the exact same samples as this reference. High abundant sequences found by amplicon sequencing give us a very precise ‘seed’ for each 16S sequence, and by carefully tuning the collection of metagenome reads matching these seeds, we were able to reconstruct a substantial number of 16S, to an adequate length and within a reasonable precision. Seemingly, the results reveal that METASEED have tremendous improvement to existing methods when it comes to high-precision re-construction of many and potentially novel 16S. Moreover, our strategy presents potentially new avenues for reconstructing the 16S rRNA gene from short-read amplicons and metagenome data.

Our approach is most useful when exploring environmental samples, where new taxa are likely to be found even among the more abundant ones. The long-read technology, otherwise providing excellent metagenome data for 16S reconstruction, is not as useful here due to highly fragmented DNA in such samples. By our proposed approach, we believe we have a tool to begin mapping the full-length 16S landscape of these environments as well.

## Methods

### Data

In this study, we use two types of data, one simulated and the other comprised of samples collected from the seafloor sediment environment.

The simulated data were all a mix of 100 genomes from 100 different species typically found in sediments. The genome information of these 100 species is available in Additional file 1, table S2. Their abundances were sampled from an exponential distribution, independently for each sample. We made 20 samples with low diversity (most abundant species dominate) and 20 high diversity samples (more equal abundances). Using the software ART version 2016.06.05, we generated simulated Illumina HiSeq reads with a length of 150bp for the shotgun samples [18]. For the corresponding 16S rRNA amplicon data we used the software InSilicoSeq version 1.5.4 to mimic Illumina MiSeq sequencing with 300bp reads [19]. The header-line of every simulated read contained information about which genome it came from, making it possible to reveal the reality of the read assignment later.

The DNA extraction and amplicon sequencing of the sediment samples have been done as described in the previously published dataset [20]. In brief, the DNA was extracted using the MagAttract PowerSoil DNA KF Kit (Qiagen). Sequencing of the V3-V4 region of the 16S rRNA gene was performed on a MiSeq platform (Illumina, CA, USA). In total 286 samples were subject to 16S sequencing, but, as described below, only a subset of 24 samples were selected for shotgun sequencing as well. The shotgun sequencing protocol stated in [20] was used here. The 24 samples were sequenced on a Novaseq 6000 platform at the Novogene, UK facility.

### Metagenome assembly

To see how easy it is to get 16S sequences directly from assembled contigs, the shotgun metagenomic reads (simulated and sediment data) were pre-processed (filtering, adapter-trimming merging of overlapping reads etc.) using the BBmap software version 1.5.4 [21]. All reads were assembled using metaSPAdes version 3.15.3 [22] with default parameters. Finally, the 16S rRNA genes in the contigs were detected using Barrnap version 0.9-foss-2018b [23]. In addition, for the comparison to above approach, as well as our own METASEED, the MATAM tool was used to reconstruct full length 16S rRNA genes directly from the shotgun reads. All the 16S sequences generated by any approach were subjected to chimera check from VSEARCH.

### The METASEED approach

#### The seeds

From the 16S data we typically find a set of sequence variants, each representing a taxon (OTU, ASV etc.) in the sample. These are the *seeds* in our procedure, and there are several pipelines for obtaining these, e.g. VSEARCH version 2.22.1 [24] or dada2 [25]. The classical 97% identity clustering has an advantage of resulting in seeds being distinctly different from each other, but due to the rather large ‘radius’ of 3% identity may very well represent more than one taxon. On the other hand, denoising like dada2 results in highly specific seeds, but these may originate from the same genome, due to differing 16S variants within the genomes. As a compromise we have settled on clustering the 16S reads at 99% identity, which is a radius that is wide enough to include all variants within a genome. The 99% radius will also include roughly 90% of all 16S variants within a species. In the supplementary material Fig. S2, we present how the current set of RefSeq genomes support this choice.

Once we generated OTUs from all samples, we set aside all the OTUs that matched with the full-length 16S sequences of the SILVA database, leaving us with a collection of pieces of 16S sequences from which we want to reconstruct the full-length version. With our V3-V4 primers, these OTU sequences are around 400–450 bases long. Next, we selected a subset of samples for full metagenome sequencing to get reads that overlaps and extend the OTU sequences. We computed the relative abundance for these OTUs. Next, we identified the samples where these OTUs were highly abundant. How many samples to select for metagenome sequencing is largely a question of resources. For a fixed number of n samples, we first selected the samples in which the top n abundant OTUs were found, and if this did not result in n samples (two OTUs may be ‘most’ abundant in the same sample), we proceeded down the list of OTUs until we reached n samples. This procedure ensures we end up with exactly n samples where we have at least one unknown OTU with a large abundance in each of them. These OTUs are the seeds we now pursue. The OTUs and the read count table is available in additional files 2 and 3 respectively.

#### Collecting metagenome reads

A key step in the reconstruction of the 16S gene is to collect reads from the metagenomes in a proper way. To gain insights on this, we conducted a study on simulated data. When simulating the metagenome reads, every read was given a header indicating which genome it comes from. In this way we could determine exactly if a collected read comes from the same genome as the seed it matches.

Given a sample, and its corresponding set of 16S seeds, the metagenome reads where aligned to the seeds in a two-step procedure. First, all metagenome reads were aligned to the seeds by bowtie2 version 2.5.0 in ‘local’ mode, and all read pairs where at least one mate had a match to a seed were collected using samtools version 1.9 [26]. Next, these reads were aligned to the seeds again using blastn version 2.12.0 [27], in order to get more details on how exactly they aligned. The initial usage of bowtie2 was exclusively for the purpose of speed, as the alignment process with blastn is considerably slower. From the blastn alignments we tested several strategies for collecting reads, based on various degrees of strictness. More specifically, this means looking for thresholds related to sequence identity, alignment lengths and how uniquely a read pair aligns to one or more seeds. For some given thresholds, we collected metagenome read pairs allocated to the various seeds, i.e. one fastq file-pair for each seed.

It should be noted that, even though reads are collected independently for each sample, the reads collected for the same seed should be merged into a single fastq file-pair after the collection is complete. For the simulated data we did not do this, but instead did the assembly (see below) separately for each sample, just to see how good/bad the choice of thresholds was with respect to reconstructing the 16S sequence.

#### Seeded assembly

**Fig. 8:**
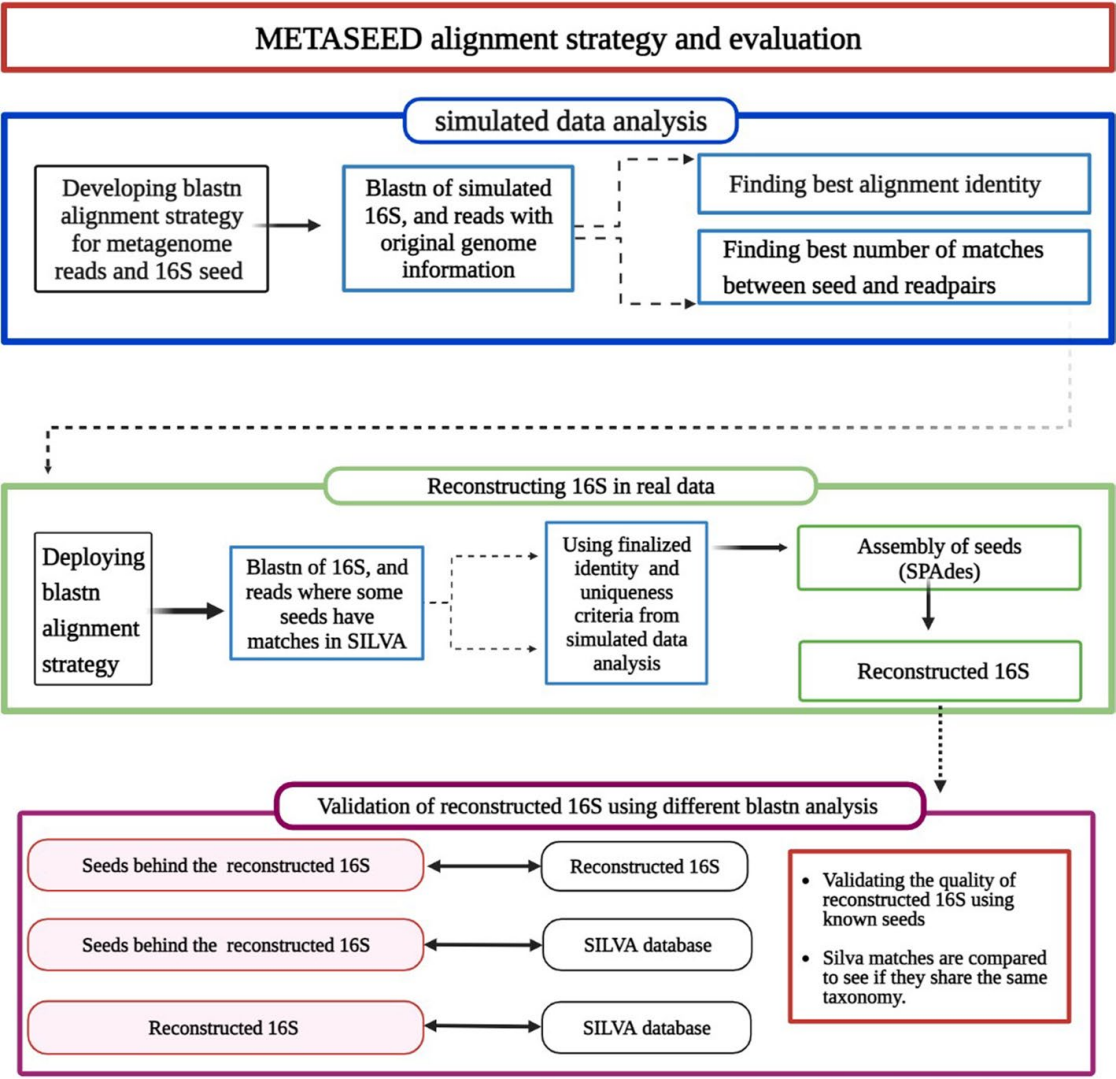
Steps involved in developing and evaluating METASEED's alignment strategy. The simulated data was analyzed to develop blastn alignment approach for seeds and metagenome reads which involved trials with varying identities and the number of matches allowed between the seed and read pairs (blue box). The METASEED strategy is then employed for the real data (green box). The seeds with matches in SILVA are then used to validate the quality of reconstructed 16S from the real data (purple box). Further, the matches of seed and reconstructed 16S in SILVA were compared for any possible similarities. Created with BioRender.com

During the preceding steps, in effect the reads have been binned before the assembly, having a pair of fastq-files with metagenome reads for each seed sequence. Thus, a straightforward assembly, as if reads where from a single genome, was used to re-construct the 16S for each seed separately. We used the SPAdes version 3.15.3 software [28] for this, and also include the seed sequence itself as a ‘trusted contig’ input to the assembly software, since SPAdes has his option. The quality of the reconstructed 16S in the real data was evaluated using different blast analyses. Figure 8 illustrates the various steps in previously mentioned METASEED alignment strategy and evaluation.

## Comparing METASEED to existing tools

As of our current understanding, no other tools specifically target the reconstruction of completely unknown 16S rRNA gene sequences while utilizing both amplicon and whole-genome sequencing (WGS) data. However, existing tools such as EMIRGE and MATAM rely on a reference set of previously known 16S sequences, to reconstruct the 16S found in WGS data. The 16S were reconstructed from the simulated data discussed in the data section, employing these three tools. Since MATAM sets a cutoff at 500 base pairs, only reconstructed sequences that met this threshold were taken into consideration. For each method, the reconstructed 16S from each sample that had at least 99% identity in VSEARCH alignment to the actual full-length 16S sequences was considered a successful reconstruction.

### Supplementary Information

**Additional file 1: Table S2**. The tables contains the genome id and organism name of the 100 species used for the simulated analysis.

**Additional file 2**. 100 centroid sequences that was used for the METASEED analysis.

**Additional file 3**. Read count for 100 centroid sequences that was used for the METASEED analysis.

## Data Availability

METASEED codes: https://github.com/MelcyPhilip/METASEED. To test the METASEED method, download METASEED.tar.gz from https://arken.nmbu.no/~pmelcy/share/METASEED/, untar and read its README.html on how to run the procedure on the small sample data set provided. The raw sequencing data are available in the NCBI SRA database under the accession # PRJNA733024 and #PRJNA1080016. The simulated data can be provided on request to the corresponding author.
